# The Role of IL-17 Promotes Spinal Cord Neuroinflammation via Activation of the Transcription Factor STAT3 after Spinal Cord Injury in the Rat

**DOI:** 10.1155/2014/786947

**Published:** 2014-04-30

**Authors:** Shaohui Zong, Gaofeng Zeng, Ye Fang, Jinzhen Peng, Yong Tao, Keke Li, Jingmin Zhao

**Affiliations:** ^1^Department of Spine Osteopathia, The First Affiliated Hospital of Guangxi Medical University, Nanning, Guangxi 530021, China; ^2^College of Public Hygiene of Guangxi Medical University, Nanning, Guangxi 530021, China; ^3^Graduate School of Guangxi Medical University, Nanning, Guangxi 530021, China; ^4^Department of Osteopathia, The First Affiliated Hospital of Guangxi Medical University, No. 22 Shuangyong Road, Nanning, Guangxi 530021, China

## Abstract

*Study Design.* In this study, we investigated the role of IL-17 via activation of STAT3 in the pathophysiology of SCI. *Objective.* The purpose of the experiments is to study the expression of IL-17 and related cytokines via STAT3 signaling pathways, which is caused by the acute inflammatory response following SCI in different periods via establishing an acute SCI model in rat. *Methods.* Basso, Beattie, and Bresnahan hind limb locomotor rating scale was used to assess the rat hind limb motor function. Immunohistochemistry was used to determine the expression levels of IL-17 and p-STAT3 in spinal cord tissues. Western blotting analysis was used to determine the protein expression of p-STAT3 in spinal cord tissue. RT-PCR was used to analyze the mRNA expression of IL-17 and IL-23p19 in the spleen tissue. ELISA was used to determine the peripheral blood serum levels of IL-6, IL-21, and IL-23. *Results.* Compared to the sham-operated group, the expression levels of IL-17, p-STAT3, IL-6, IL-21, and IL-23 were significantly increased and peaked at 24 h after SCI. The increased levels of cytokines were correlated with the SCI disease stages. * Conclusion.* IL-17 may play an important role in promoting spinal cord neuroinflammation after SCI via activation of STAT3.

## 1. Introduction

The spinal cord is a component of the central nervous system, which connects the brain and peripheral nervous system. Spinal cord injury (SCI) is a severe traumatic injury to the central nervous system that usually results in sensation disability and paralysis, and at present, there is still no effective therapy [1]. Pathologically, SCI can be divided into primary injury and secondary injury phases. Tissue damage and bone fractures directly resulting from violent forces belong to the primary injury phase, which initiates serial inflammation lesion known as secondary injury. Primary injury is unexpected and thus the only part that may be interposed to protect or rescue the cord is the secondary injury phase [2]. However, the mechanisms of underlying secondary injury are still not fully defined. In addition, present studies have demonstrated that the pathogenesis of neurological diseases is associated with autoimmune or immune-related inflammatory injury [3].

IL-17 is an important proinflammatory factor, which is mainly produced by Th17 cells and plays an important role in inflammation [4]. IL-6 is a key regulator of IL-17 production, whereas it inhibits the regulatory T cells [5]. IL-23 plays an essential role in various inflammatory diseases, which can promote IL-17 production as well as IL-22 and IL-17 produced by Th17 cells. Moreover, IL-21 together with transforming growth factor-**β** potently stimulates IL-17 production [6]. In addition, ROR*γ*t and STAT3 are two critical transcription factors which can mediate IL-17 production and Th17 differentiation.

It has been recently demonstrated that IL-17 is critical for autoimmune disease [7] and the data clearly support the notion that both anti-inflammatory (Th2 or Tregs) and proinflammatory (Th1 or Th17) T cells were activated in the context of a sterile SCI [8]. Thus, investigation of the function of IL-17 and IL-17 related cytokines in spinal cord injury may reveal the pathogenesis of these diseases and provide a valuable insight for the treatment of spinal cord injury-related diseases.

## 2. Materials and Methods

### 2.1. Ethics Statement

The protocol for the study was approved by the Institutional Animal Experiment Committee of Guangxi Medical University (Nanning, China; permission numbers: 2012, KY-E-006). All animal experimentation was performed in accordance with the guidelines for the ethical care and treatment of rats from the European Community Guidelines (EEC Directive of 1986; 86/609/EEC).

### 2.2. Animals and Treatments

All efforts were made to minimize all animals' suffering. Seventy-five 60-day-old male Sprague Dawley rats were purchased from the Guangxi Medical College Animal Experimentation Center (certificate number SCXK GUI 2009-0002) and were used for experiments at 65 days of age (weight range: 225–275 g). A total of 75 adult male SD rats were divided into two groups, the sham-operated group and the SCI group. Rats in the SCI group were randomly placed into the following subgroups: 1 h, 24 h, 48 h, and 72 h (*n* = 15). SCI (40 g × cm) was induced using the weight drop technique, as previously reported [9]. Rats were anesthetized with an intraperitoneal injection of chloral hydrate (80 g/L in saline solution; 0.4 mL/100 g body weight) followed by midline skin incision at T5-T12 and paravertebral muscle dissection under sterile surgical conditions. Laminectomy was performed at the T9-T11 vertebral level with the assistance of a surgical microscope. A 5 g weight was dropped from a height of 8 cm onto the dura-exposed spinal cord. The SCI was performed at the T10 level. The absorbable gelatin sponge was placed at the site of the SCI to hemostasis and was sutured to the vertebral column. And then, the skin and musculature were sutured. Laminectomy was performed on the sham-operated group without SCI. After surgery, all rats were housed in warm cages with food and water supplied* ad libitum*. The rats were monitored daily. Rats that underwent SCI received specialized care consisting of manual bladder expression twice daily and cleansing for the duration of the experiment.

### 2.3. Behavioral Testing

Rats from the SCI group and sham-operated group were assessed for hind limb motor function at 1 h, 24 h, 48 h, and 72 h after injury by two blinded observers using the Basso, Beattie, and Bresnahan (BBB) hind limb locomotor rating scale test. Afterwards, each rat was placed in an open field and was scored by two blind observers after an observation period of 5 min.

### 2.4. Tissue Preparation

The peripheral blood was collected from each group, which consisted of 15 rats. Rats from the SCI group were sacrificed at the same daytime 1, 24, 48, and 72 h after operation using deep anesthesia with an overdose of chloral hydrate (10%). The rats were transcardially perfused with approximately 200 mL of 4% paraformaldehyde in 0.01 M PBS, and a 10 mm cord segment that included the site of the SCI at T10 was harvested. The spleens were removed aseptically as fresh specimens for measurement before perfusion with PFA.

### 2.5. Hematoxylin-Eosin Staining and Immunohistochemistry

Spinal cord tissue segments were cut longitudinally, fixed in 10% paraformaldehyde for 24 h, embedded, and sectioned at 5 *μ*m thickness. Hematoxylin-eosin staining was used to observe the morphological changes. Immunohistochemical staining was performed by the streptavidin-biotin complex methods. The sections were stained by the streptavidin-biotin complex kit (Boter, Wuhan, China). According to the streptavidin-biotin complex kit manufacturer's instructions, we have chosen the following primary monoclonal antibodies: IL-17 (Santa Cruz Biotechnology, USA) at a working concentration of 5 *μ*g/mL and p-STAT3 (Santa Cruz Biotechnology) at a working concentration of 5 *μ*g/mL. To assess the expressions of IL-17 and p-STAT3, we measured 10 samples in one section chosen at random from each spinal cord. Digital images of IL-17 and p-STAT3 immunohistochemistry were obtained using a light microscope (Eclipse E800, Nikon, Japan). The Software Image-Pro Plus Version 5.0 (Media Cybernetics, Bethesda, MD, USA) was used to measure the integrated optical density (IOD).

### 2.6. Western Blotting Analysis

After the spinal cord tissues were harvested, total tissue protein was extracted using RIPA lysate containing PMSF and 1% protease inhibitor. The protein concentration of the spinal cord tissue extract was determined using the BCA method and stored at −70°C. After an equivalent amount of the sample was added, the proteins were separated by 8% and 12% SDS-PAGE gel electrophoresis and transferred onto nitrocellulose membranes. The membranes were blocked in Tris-buffered saline and Tween 20 (TBST, pH 7.6) containing 5% FBS for 1 h and thereafter incubated at 4°C overnight with the following primary antibodies: p-STAT3Tyr705 (1 : 2000 dilutions) and GAPDH (1 : 10000 dilutions). After several washes with TBST, the membranes were probed with secondary antibodies (1 : 15000 dilutions) at room temperature for 2 h. The gray value of the protein bands was detected using image analysis system. GAPDH was used as a loading control.

### 2.7. Semiquantitative Detection of IL-17 and IL-23p19 mRNA

Total RNA was extracted from homogenized spleen tissues using TRIZOL reagent (Invitrogen, USA) and was used to synthesize cDNA with an RT kit (Ferma, USA). According to the manufacturer's protocol, reverse-transcription PCR (RT-PCR) was performed with first-strand cDNA synthesized with 1 *μ*g of total RNA and oligo d (T) 18 primers. The primers for the RT-PCR assays for IL-17 and IL-23p19 were designed using Primer Premier 5.0 (Table 1). Rat **β**-actin, a reference gene, was used to normalize each sample and each gene. The prepared cDNA was used for PCR amplification with the above primers under the following conditions: preheating at 94°C for 3 min, denaturing at 94°C for 30 sec, annealing at 60°C (IL-17) or 58°C (IL-23) for 30 sec, and extension at 72°C for 40 sec. The reaction was repeated for 35 cycles followed by incubation at 72°C for 10 min. The PCR products were analyzed using electrophoresis on a 2% agarose gel electrophoresis and visualized with 0.5 mg/mL ethidium bromide staining and ultraviolet transillumination. The resulting bands were observed and imaged under ultraviolet light and then measured using the Digital Gel Imaging Analyst (Nikon 990-Doc 1000, USA). The density was determined for each sample PCR product including the positive control. The background density was subtracted from each band and the relative values of IL-17 and IL-23p19 mRNA were calculated using **β**-actin mRNA as a standard. The PCR products were blasted in the NCBI Blast bank and sequenced by Sangon Biotech Co., Ltd (Shanghai, China).

### 2.8. Cytokine Analysis by ELISA

For plasma preparation, the peripheral blood was centrifuged at 3000 ×g for 20 min. Then, the cell-free upper phase was collected and stored at −80°C. The concentration of IL-6, IL-21, and IL-23 was measured using ELISA kits (Huamei, Wuhan). Briefly, 96-well microtiter plates and other reagents were incubated at room temperature prior to use for 30 min. Next, 100 *μ*L of standard and sample were added per well and incubated at 37°C for 2 h. The liquid was removed from each well without washing, and 100 *μ*L of biotin-antibody was added to each well and incubated at 37°C for an additional 1 h. After washing, unbound antibodies were washed off, followed by the addition of avidin-HRP (1 : 1000 dilutions). The plates were incubated at 37°C for 1 h. Finally, the color reaction was developed by incubation with a TMB substrate and the enzyme reaction was terminated with 50 *μ*L of stop solution. The OD was read at 450 nm using an ELISA plate reader (Bio-Rad) and then the standard curves were established to quantify the amount of specific cytokines.

### 2.9. Statistical Analyses

All data were presented as the mean ± SEM. Statistical analyses were performed using SPSS16.0 (SPSS Company, USA). One-way ANOVA followed by the LSD multiple-range test was used to analyze the differences between groups. Spearman's correlation analysis was used to assess the correlations between normally distributed variables. *P* < 0.05 (two-tailed) was used as the level of statistical significance.

## 3. Results

### 3.1. BBB Scores

We used the BBB locomotor rating scale to assess neurological function at 1 h, 24 h, 48 h, and 72 h after injury. The mean BBB scores (Table 2) of the SCI group were lower than the sham group at 1 h, 24 h, 48 h, and 72 h after injury (*P* < 0.05). The mean BBB scores in the SCI group increased from 1.41 at 24 h to 4.51 at 72 h (Table 2). The sham group was not damaged to nerve function so their BBB scores were full marks and the SCI 1 h group was unconscious so their BBB scores were zeros. Neurologic function in the SCI group corresponded to an improvement from rarely movement of two joints to extensive movement of two joints and slight movement of one joint. The BBB scores reflected neurological function improvement from slight movement of three joints to weight-supported plantar steps and frequent coordination.

### 3.2. Hematoxylin-Eosin Staining

**Figure 1:**
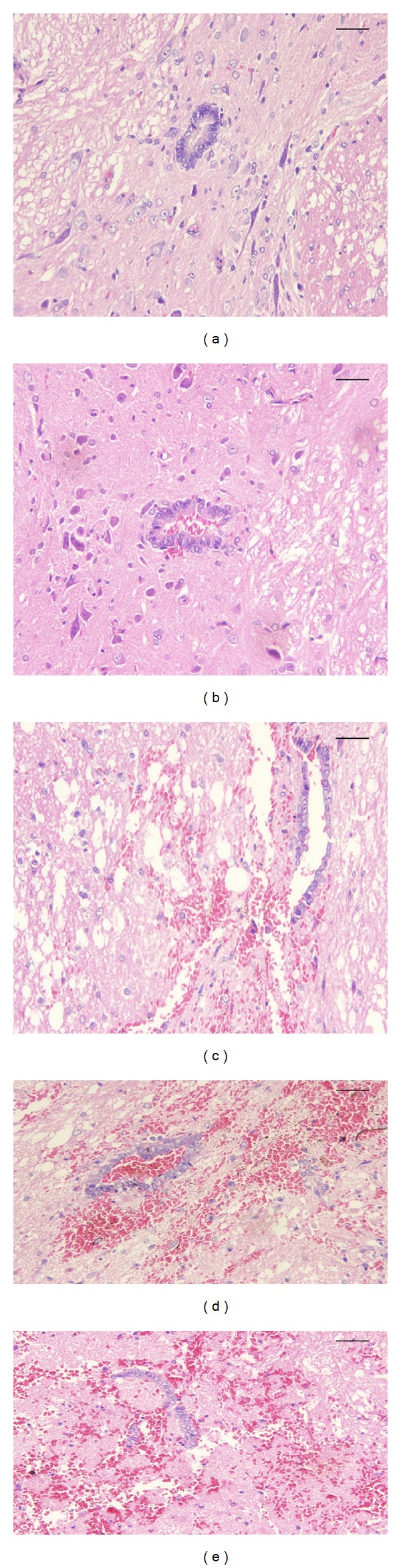
The severity of SCI was evaluated using hematoxylin-eosin staining (original magnification ×400). The spinal cord tissues were normal in the sham-operated group (a). The spinal cord demonstrated obvious sign of compression following SCI and the organization structure was destroyed as observed in (b). Next, the degree of inflammation gradually increased. A few scattered small foci of hemorrhage from small veins and capillaries and nerve cells are swollen and necrosis was observed at 24 h (c). The severity of the nerve cell necrosis and interstitial inflammatory cell infiltration were markedly increased at 48 h (d). Extensive hemorrhage and nerve cell necrosis was observed in at 72 h (e). Bar equals 50 *μ*m.

In the SCI group, the structural integrity of the spinal cord was damaged and the weight hit locations showed a strong compression phenomenon. HE staining revealed a progressive increase in inflammatory cell expression at sites of injury and neuronal damage from 1 to 72 h after surgery. In the SCI 1 h group, the hemorrhage from the capillaries, some neurons of edema, and dead neurons were observed in the spinal cord but were comparatively less compared to the spinal cord injured rats at 24 h. In the SCI 48 h to 72 h group, progressive hemorrhage and enhanced inflammatory infiltration of the tissue architecture in the white and central gray matter, more dead neurons, and apoptotic bodies were observed in the injured cord around the lesion. In addition, the apoptotic area and spinal cord cavity formation were observed in the lesion at 72 h after injury. All neurons were normal in the sham-operated group (Figure 1).

### 3.3. Immunohistochemistry

**Figure 2:**
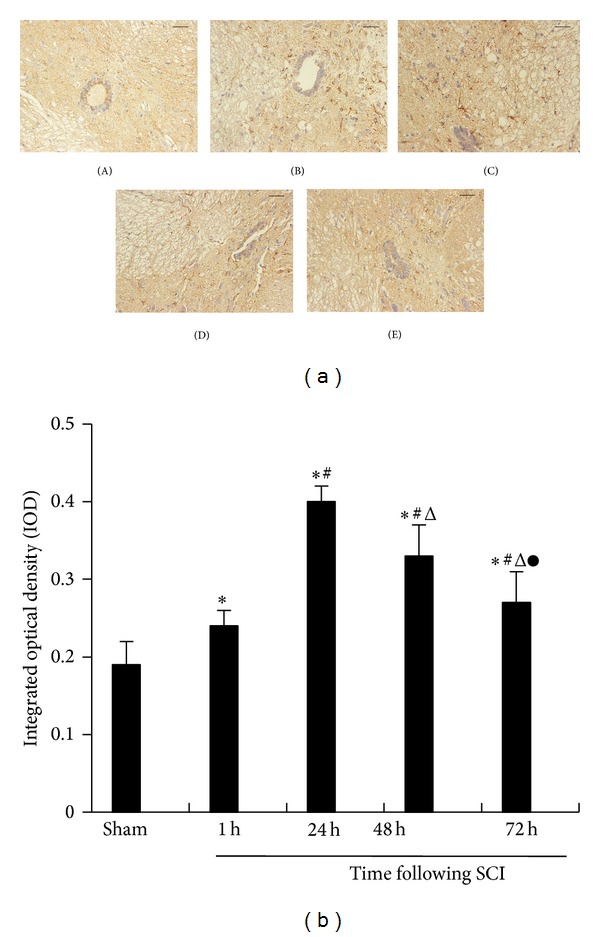
(a) Representative IL-17 immunohistochemistry images in spinal cord tissue (brown granules, original magnification ×400). The sham-operated groups (A) and SCI group at 1 h (B), 24 h (C), 48 h (D), and 72 h (E). A digitized image shows labeled IL-17 immunohistochemistry in tissue. The gray value of the IL-17 immunohistochemical expression was assessed. IL-17 expression significantly increased in the rat SCI model group and peaked at 24 h. Bar equals 50 *μ*m. (b) Morphometric quantitate of IL-17 protein expression in spinal cord tissues. The IOD in different groups was 0.19 ± 0.03, 0.24 ± 0.02, 0.40 ± 0.02, 0.33 ± 0.04, and 0.27 ± 0.04, respectively. Representative spinal cord tissue sections from the sham-operated group (*n* = 15) and SCI groups at 1 h (*n* = 15), 24 h (*n* = 15), 48 h (*n* = 15), and 72 h (*n* = 15) rats. The data are presented as the mean ± the SE. *P < 0.05 versus sham, ^#^ *P* < 0.05 versus 1 h, ^△^ *P* < 0.05 versus 24 h, and ^●^ *P* < 0.05 versus 48 h.

**Figure 3:**
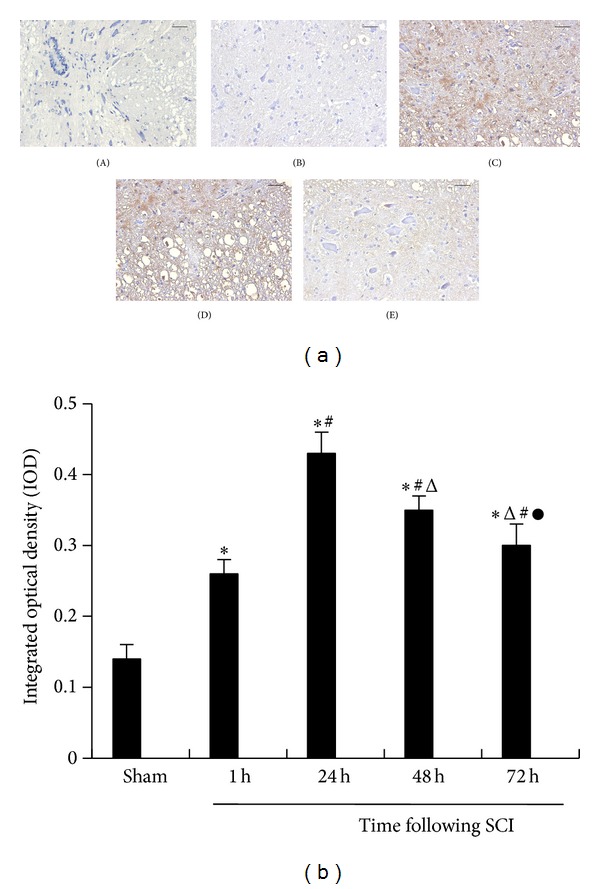
(a) Representative p-STAT3 Immunohistochemical images in spinal cord tissue (brown granules, original magnification ×400). The sham-operated group (A) and SCI groups at 1 h (B), 24 h (C), 48 h (D), and 72 h (E). The digitized image shows labeled p-STAT3 immunohistochemistry on tissue. The IOD of p-STAT3 immunohistochemistry was assessed. The rat SCI model group showed significantly increased p-STAT3 expression and peaked at 24 h. Bar equals 50 *μ*m. (b) Morphometric quantitate of p-STAT3 protein expression in spinal cord tissues. The IOD in different groups was 0.14 ± 0.02, 0.26 ± 0.02, 0.43 ± 0.03, 0.35 ± 0.02, and 0.30 ± 0.03, respectively. Representative spinal cord tissue sections from the sham-operated group (*n* = 15) and SCI groups at 1 h (*n* = 15), 24 h (*n* = 15), 48 h (*n* = 15), and 72 h (*n* = 15) rats. The data are presented as the mean ± the SE. *P < 0.05 versus sham, ^#^ *P* < 0.05 versus 1 h, ^△^ *P* < 0.05 versus 24 h, and ^●^ *P* < 0.05 versus 48 h.

We used immunohistochemistry to determine the expression of IL-17 and p-STAT3 which assessed the progression of the SCI. In the sham-operated group, IL-17 and p-STAT3 demonstrated nearly no expressions. In the spinal cord injury group, the expressions of IL-17 and p-STAT3 were gradually increased in the SCI 1 h group and peaked at 24 h after spinal cord injury. In the SCI 48 h group, the expressions of IL-17 and p-STAT3 were slowly decreased, but its expressions in the SCI 72 h group were still higher than the sham-operated group (Figures 2 and 3). In the SCI group, IL-17 and p-STAT3 integrated optical density (IOD) were higher than the sham-operated group (*P* < 0.05) (Figures 2 and 3).

### 3.4. Activation of STAT3 after SCI

**Figure 4:**
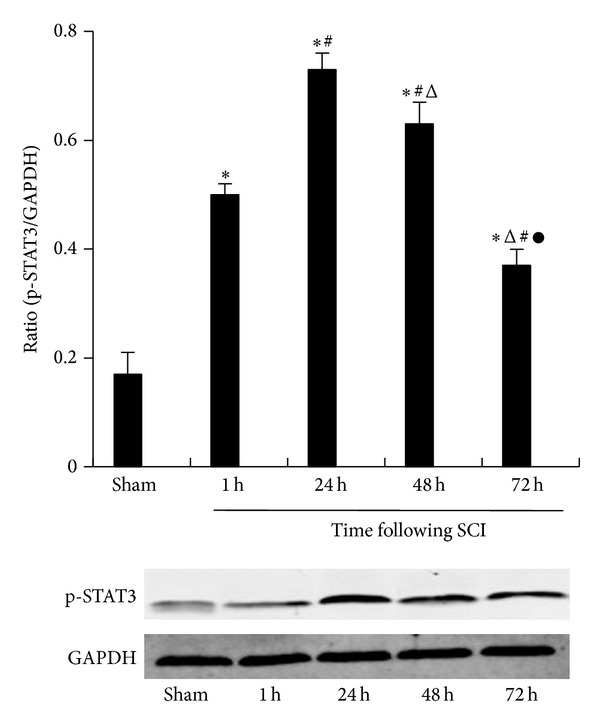
Phosphorylation of STAT3 protein levels in the spinal cord. The protein levels of p-STAT3 in the spinal cord tissues in different groups, as measured by western blotting analyses. The spinal cord tissues levels were 0.17 ± 0.04, 0.50 ± 0.02, 0.73 ± 0.03, 0.63 ± 0.04, and 0.37 ± 0.03, respectively, in the sham-operated group (*n* = 15) and SCI groups at 1 h (*n* = 15), 24 h (*n* = 15), 48 h (*n* = 15), and 72 h (*n* = 15) rats. The data are presented as the mean ± the SE. *P < 0.05 versus sham, ^#^ *P* < 0.05 versus 1 h, ^△^ *P* < 0.05 versus 24 h, and ^●^ *P* < 0.05 versus 48 h.

We used western blotting analyses to determine activation of the STAT3 signaling pathway after SCI. Antibodies against p-STAT3 detected a single protein band in the crude samples of injured spinal cord, similar to the sham-operated group in all detected groups. Densitometric analysis of p-STAT3 at Tyr705 bands revealed an increased expression after SCI immediately, peaking at 24 h and gradually decreasing thereafter, while p-STAT3 was hardly detected in control groups (Figure 4). Statistical significance was seen from 1 to 72 h after SCI compared with the control value (*P* < 0.05).

### 3.5. Different Levels of IL-17 and IL-23p19 mRNA

**Figure 5:**
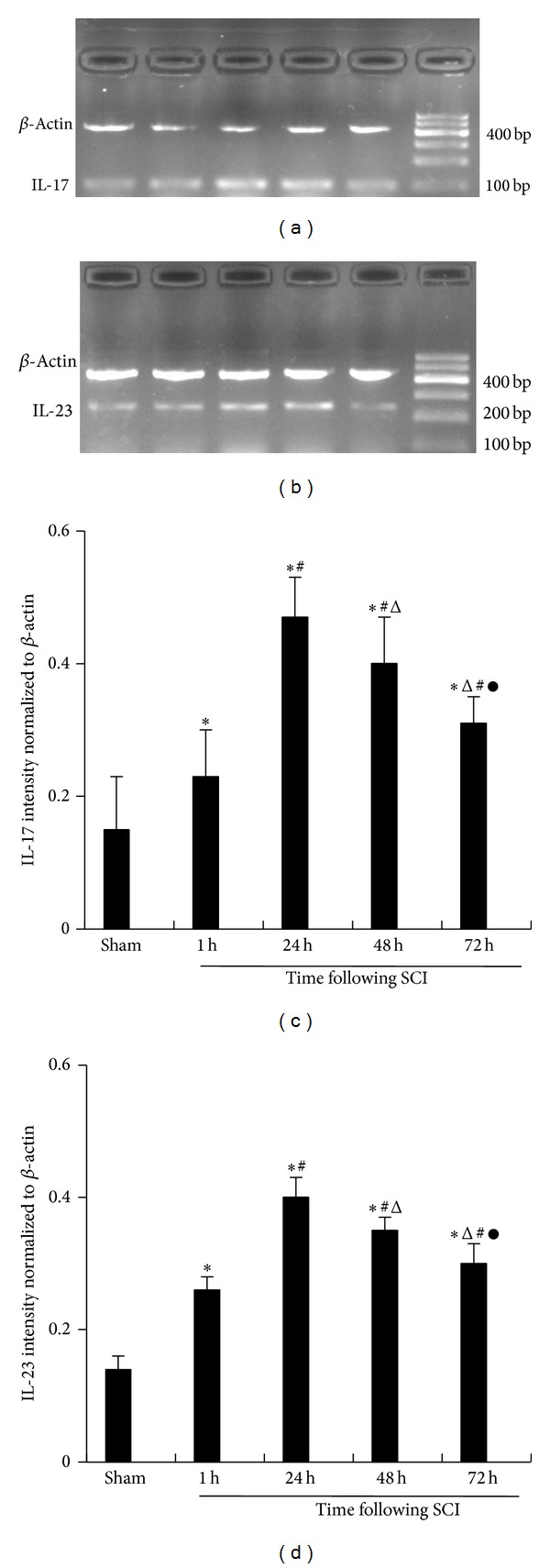
(a) and (b): IL-17 and IL-23p19 mRNA transcription in spleen tissue. Representative images showed semiquantitative RT-PCR for IL-17 and IL-23p19 transcription sham. The sham-operated group; 1 h. The 1 h group of SCI; 24 h. The 24 h group of SCI; 48 h. The 48 h group of SCI; 72 h. The 72 h group of SCI. (c) and (d): densitometric quantification of IL-17 and IL-23p19mRNA expression in spleen tissues. Densitometric quantification of PCR bands showed that, among the IL-17 and IL-23p19, the sham-operated group, 1 h group of SCI, 24 h group of SCI, 48 h group of SCI, and 72 h group of SCI, the mRNA expression was significantly difference between each groups and peaked at 24 h. The data are presented as the mean ± the SE. *P < 0.05 versus sham, ^#^ *P* < 0.05 versus 1 h, ^△^ *P* < 0.05 versus 24 h, and ^●^ *P* < 0.05 versus 48 h.

RT-PCR was used to detect the expressions of IL-17 and IL-23p19 mRNA and its intensity was normalized against **β**-actin. For IL-17, the results in the SCI group from 1 h to 72 h were 0.23 ± 0.07, 0.47 ± 0.06, 0.40 ± 0.07, and 0.31 ± 0.04. The results in the sham group were 0.15 ± 0.08. The IL-17 mRNA was clearly upregulated in rats with SCI at 1 h and peaked at 24 h (*P* < 0.05). For IL-23p19 mRNA, the expression of spleen mRNA in the SCI group from 1 h to 72 h was 0.25 ± 0.05, 0.54 ± 0.07, 0.47 ± 0.09, and 0.39 ± 0.07. Data in the sham group was 0.14 ± 0.06. The IL-23p19 mRNA was clearly upregulated in rats with SCI at 1 h and peaked at 24 h (*P* < 0.05) (Figure 5).

### 3.6. Increased IL-6, IL-21, and IL-23 Concentrations in the Periphery following SCI

**Figure 6:**
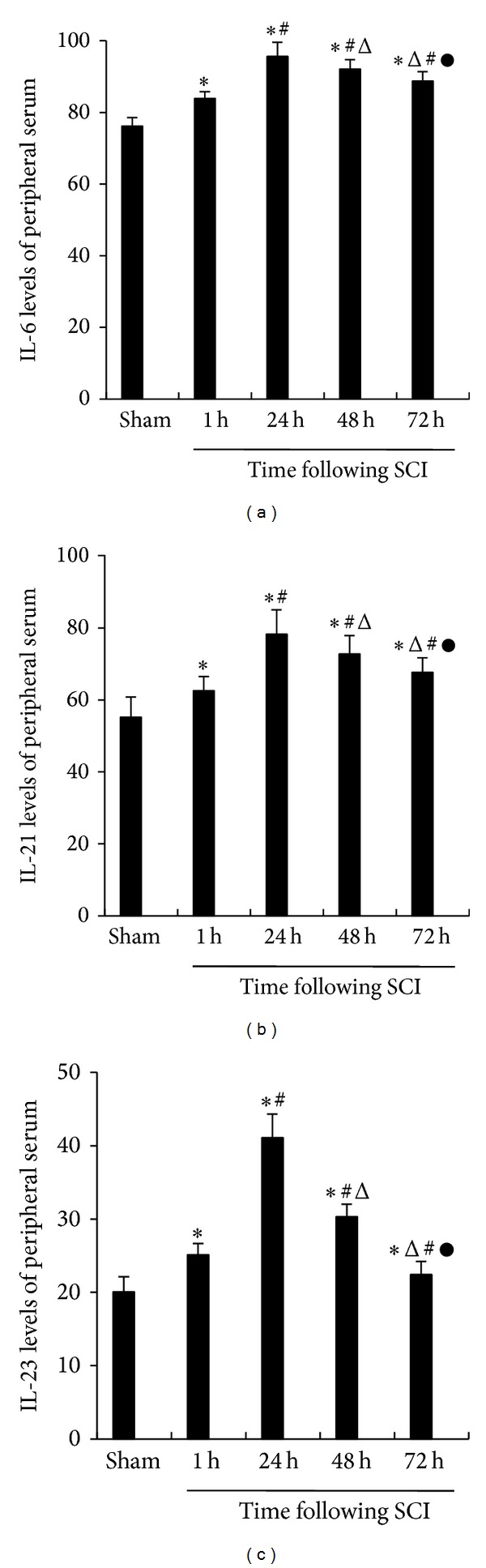
The IL-6, IL-21, and IL-23 levels of peripheral serum. The levels in the IL-6, IL-21, and IL-23 in the different groups, measured by ELISA. The IL-6 levels were 76.18 ± 2.36, 83.93 ± 1.85, 95.65 ± 3.97, 92.10 ± 2.64, and 88.81 ± 2.58. The IL-21 levels were 55.22 ± 5.59, 62.58 ± 3.91, 78.25 ± 6.82, 72.73 ± 5.18, and 67.65 ± 4.02. The IL-23 levels were 20.05 ± 2.07, 25.15 ± 1.51, 41.12 ± 3.19, 30.33 ± 1.69, and 22.42 ± 1.78, respectively, in the sham-operated group (*n* = 15), 1 h group of SCI (*n* = 15), 24 h group of SCI (*n* = 15), 48 h group of SCI (*n* = 15), and 72 h (*n* = 15) group of SCI. The data are presented as the mean ± the SE. *P < 0.05 versus sham, ^#^ *P* < 0.05 versus 1 h, ^△^ *P* < 0.05 versus 24 h, and ^●^ *P* < 0.05 versus 48 h.

Concentrations of peripheral serum IL-6, IL-21, and IL-23 were measured by ELISA. There were increased expressions of IL-6, IL-21, and IL-23 in the SCI group from 1 h to 72 h and peaked at 24 h compared to the sham group (Figure 6).

### 3.7. Correlation between STAT3 and IL-17 Related Cytokines in the SCI Environment in the Rat Model

Our study succeeded in demonstrating the statistical correlation between p-STAT3 and Th17 cell related cytokines. Immunohistochemistry demonstrated that p-STAT3 concentration showed a positive correlation with the level of IL-17 (*r* = 0.5415, *P* = 0.0054) in spinal cord tissue following SCI. RT-PCR demonstrated that IL-17 concentrations showed a positive correlation with the level of IL-23p19 (*r* = 0.6213, *P* = 0.0135) in spleen tissue following SCI. Moreover, IL-17 reached statistical significance with the level of IL-6 (*r* = 0.4735, *P* = 0.026), IL-21 (*r* = 0.5219, *P* = 0.0419), or IL-23 (*r* = 0.5017, *P* = 0.0387) following SCI.

## 4. Discussion

Traumatic spinal cord injury results in the disruption of neural and vascular structures, which leads to inflammation and the initiation of secondary injury events defined secondary spinal cord injury. Indeed, many experimental SCI models have indicated that traumatic spinal cord injury is associated with long-lasting inflammation, which parallels the force of the insult and the severity of the functional deficit [10]. Currently, there is no treatment available for SCI. Because hemorrhage and subsequent inflammation result in a worse neurological outcome and larger lesion following SCI, it is essential to study the secondary spinal cord injury to reduce the secondary damage in order to preserve motor function [11]. An enhanced understanding of inflammation will preserve spinal cord tissue and neurons better, which may correct biochemical disorder at the immediate and distal sites of spinal cord injury, including the imbalance of inflammatory molecules and the loss in nerve function, thereby reducing secondary spinal cord degeneration [12]. Recent studies have identified IL-17 (Th17) originated from the subset of T cells, which plays a predominant role in the pathogenesis of experimental autoimmune encephalomyelitis [13] and autoimmune arthritis [14, 15]. While most nervous system disease pathogeneses are associated with autoimmune or immune inflammatory injury [3], the data clearly support the notion that both anti-inflammatory (Th2 or Tregs) and proinflammatory (Th1 or Th17) T cells were activated in the context of a sterile SCI [8]. Importantly, recent clinical trials with short duration IL-17 antagonistic therapy in established rheumatoid arthritis (RA) have provided the direct evidence in pathological role of IL-17 in RA and indicated that blockade of IL-17 in humans may represent a valid therapeutic approach [16]. Thus, we investigated whether the IL-17 and IL-17 related cytokines can affect secondary inflammatory reaction after spinal cord injury, which may provide a valid therapeutic approach for SCI.

In this study, we firstly observed that aberrant STAT3 activation and IL-17 related cytokines' overexpression were involved in SCI pathogenesis which may potentially aggravate this injury. The most common type of SCI in the clinical practice is contusive spinal cord injury; the weight drop rat model of SCI described by Allen is the most appropriate model to assess the acute SCI [17]. In this model, compared to the sham-operated group, hematoxylin-eosin staining results in the SCI group exhibited marked neurons and organizational structure damage in the spinal cord tissues. Furthermore, enhanced inflammatory infiltration and neuronal apoptosis were observed in the spinal cord tissue during SCI. Our previous studies have demonstrated that IL-1 induces secondary inflammation of SCI at 12 h [9]. These data were consistent with the neuronal apoptosis observed in secondary SCI in the rat model and the peak times were from 24 h to 72 h [18]. In addition, compared to the sham-operated group, the protein expression level of IL-17 in the SCI group was higher and peaked at 24 h. Consistently with these results, the mRNA expression level of IL-17 was increased after SCI and peaked at 24 h in spleen tissue samples. Totally, these data indicated that IL-17 may play a very important role in the pathophysiology of SCI.

Phosphorylation of STAT3 is considered critical for constraining IL-17 production [19]. IL-17 involved in the pathophysiology of inflammatory diseases required specific cytokines and transcription factors for their differentiation. Specifically, IL-6 and TGF-**β** are recognized as crucial factors for T cells differentiation [20, 21]. STAT3 is known as a primary factor in the downstream signaling of IL-6, the phosphorylation of which induces proinflammatory gene expression [22, 23]. Furthermore, Treg cell differentiation can be suppressed by IL-6 via inhibition of the expression of Foxp3 in a STAT3-dependent manner and thereby contributes to immune pathology [24, 25]. Therefore, we assessed the phosphorylation of STAT3 induced IL-17 activation of CD4 T cells following SCI to further study the underlying mechanisms of IL-17/STAT3 balance. Interestingly, the SCI group demonstrated increased protein levels of p-STAT3 compared to the sham-operated group and peaked at 24 h. In addition, phosphorylation of STAT3 expression was higher in the SCI group and peaked at 24 h compared to the sham-operated group as assessed using immunohistochemistry. These data were consistent with the significant characteristics of the JAK-STAT signaling pathway, where metabolism quickly starts and disappears [26]. Under normal physiological conditions, JAK-STAT will momentarily be phosphorylated and this activation state only lasts for a few minutes to several hours [27]. Moreover, we observed that the SCI group demonstrated an obvious increase in the phosphorylation of STAT3. As Figures 2 and 3 are shown, the positive expression of IL-17 and p-STAT3 were both concentrated in the spinal cord neurons and astrocyte cells. Therefore, IL-17 and p-STAT3 are closely related with spinal cord neuroinflammation. Considering the dynamic balance between the IL-17 and p-STAT3, we further analyzed the correlation between IL-17 and p-STAT3 using Spearman's correlation analysis, which showed a positive correlation (*r* = 0.5415, *P* = 0.0009) in the immunohistochemistry results. In addition, our study on spleen tissues demonstrated a statistical correlation between p-STAT3 protein levels and IL-17 mRNA, which also showed a positive correlation (*r* = 0.5415, *P* = 0.0009). Thus, we demonstrated that the STAT3 signaling pathway was activated and induced by SCI, suggesting that activation of p-STAT3 might contribute to the effect on IL-17 expression.

The inflammatory response is an essential component of the repair process after spinal cord injury, which is associated with the upregulation of adhesion molecules, recruitment of inflammatory cells, growth factors, and cytokines. A variety of cytokines, including most interferons (IFNs), interleukins (ILs), and colony stimulating factors (CSFs), mediate their effects via the JAK/STAT pathway [28]. Currently, IL-6 was recognized to be one of the key cytokines accelerating the IL-17 production [29]. As an autocrine growth factor, IL-21 exerts critical functions in IL-17 production and induces the differentiation of IL-21-producing CD4^+^ T cells [30]. IL-23 and IL-17 are proinflammatory cytokines of the IL-23/IL-17 axis which may play a key role in the pathogenesis of autoimmune diseases [31], such as IL-17 proliferation, maintenance, and amplification [32]. To gain insight into the mechanism underlying IL-17 differentiation, IL-17 mRNA, IL-23p19 mRNA, and serum levels of IL-6, IL-21, and IL-23 were measured in all animals. Our data showed that the serum levels of IL-6, IL-21, and IL-23 were increased at 1 h after injury and peaked at 24 h. IL-17 mRNA increased the circulating levels of IL-17 and IL-23p19 mRNA and increased circulating IL-6, IL-21, and IL-23 levels. Consistent with this finding, the levels of IL-23p19mRNA expression were also increased. As a result, IL-6, IL-21, and IL-23 had a positive correlation with the increased IL-17. To a large extent, the results of our study confirmed the hypothesis that, in the neuroinflammation of SCI, IL-6, IL-21, and IL-23 are produced in large quantities. These changes are more conductive to the production of IL-17 and lead to immune disorder and promotion of SCI. Moreover, the excessive expression of IL-17 activated STAT3 signaling pathway. Therefore, an interconnected loop is formed among spinal cord neuroinflammation factors, IL-17, STAT3 signaling pathway, and IL-17 related cytokines, which could influence each other and promote the development of spinal cord neuroinflammation.

There are several limitations in our study. First, IL-17 plays a central role in the serious stage of SCI. IL-17 demonstrated increases both at the gene and at protein expression level. Thereafter, we independently detected the protein level of IL-17 and p-STAT3 using immunohistochemistry. The results demonstrated that IL-17 can induce the abnormal activation of STAT3 following SCI. Second, we focused on IL-17 and its related cytokines IL-6, IL-21, and IL-23; our study confirmed a positive correlation between IL-17 and its related cytokines. Furthermore, we did not show that IL-17 came from the major CD4^+^ T cell subset and we were unable to accomplish double staining for IL-17 and p-STAT3. In addition, when STAT3 signaling pathway is blocked, the expressions of IL-17 and p-STAT3 in spinal cord neuroinflammation after SCI still need further investigation in our next experiments.

## Figures and Tables

**Table 1:** Sequences of primers for PCR.

Molecule	Sequences (5′-3′)	Length
IL-17A	Sense: 5′-GGGAAGTTGGACCACCACCT-3′	101 bp
[GenBank: 301289]	Antisense: 5′-TTCTCCACCCGGAAAGTGAA-3′	

IL-23p19	Sense: 5′-AAAGGAGGTTGATAGAGGGT-3′	241 bp
[GenBank: 155140]	Antisense: 5′-TCTTAGTAGATCCATTTGTCCC-3′	

*β*-actin	Sense: 5′-GAGAGGGAAATCGTGCGTGAC-3′	452 bp
[GenBank: 81822]	Antisense: 5′-CATCTGCTGGAAGGTGGACA-3′	

**Table 2:** The mean BBB scores in the rat model of SCI.

The sham group		The SCI group			
		1 h	24 h	48 h	72 h
*n*	15	15	15	15	15
BBB Scores	24.00 ± 0.00	0.00 ± 0.00	1.41 ± 0.87^●^	2.76 ± 1.44^▲^	4.51 ± 1.88^★^

Data are expressed as the mean ± the SE.

^●^ *P* < 0.05, versus sham; ^▲^ *P* < 0.05 versus 24 h; ^★^ *P* < 0.05 versus 48 h (significant differences).
