# Correction to “The Role of IL‐17 Promotes Spinal Cord Neuroinflammation via Activation of the Transcription Factor STAT3 after Spinal Cord Injury in the Rat”

**DOI:** 10.1155/mi/9869465

**Published:** 2026-06-24

S. Zong, G. Zeng, Y. Fang, et al., “The Role of IL‐17 Promotes Spinal Cord Neuroinflammation via Activation of the Transcription Factor STAT3 after Spinal Cord Injury in the Rat,” *Mediators of Inflammation* 2014, no. 1 (2014): 786947, https://doi.org/10.1155/2014/786947.

In the above article, an error during figure preparation led to a duplication in Figure 3C,D. The correct Figure 3 is as follows:

Figure 3(a) Representative p‐STAT3 immunohistochemical images in spinal cord tissue (brown granules, original magnification ×400). The sham‐operated group (A) and SCI groups at 1 h (B), 24 h (C), 48 h (D), and 72 h (E). The digitized image shows labeled p‐STAT3 immunohistochemistry on tissue. The IOD of p‐STAT3 immunohistochemistry was assessed. The rat SCI model group showed significantly increased p‐STAT3 expression and peaked at 24 h. Bar equals 50 μm. (b) Morphometric quantitate of p‐STAT3 protein expression in spinal cord tissues. The IOD in different groups was 0.14 ± 0.02, 0.26 ± 0.02, 0.43 ± 0.03, 0.35 ± 0.02, and 0.30 ± 0.03, respectively. Representative spinal cord tissue sections from the sham‐operated group (*n* = 15) and SCI groups at 1 h (*n* = 15), 24 h (*n* = 15), 48 h (*n* = 15), and 72 h (*n* = 15) rats. The data are presented as the mean ± the SE.  ^∗^ *p*  < 0.05 versus sham, ^#^ *p*  < 0.05 versus 1 h, ^△^ *p*  < 0.05 versus 24 h, and ^●^ *p*  < 0.05 versus 48 h.

**Figure:**
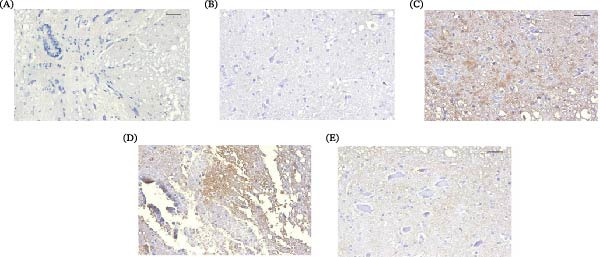
(a)

**Figure:**
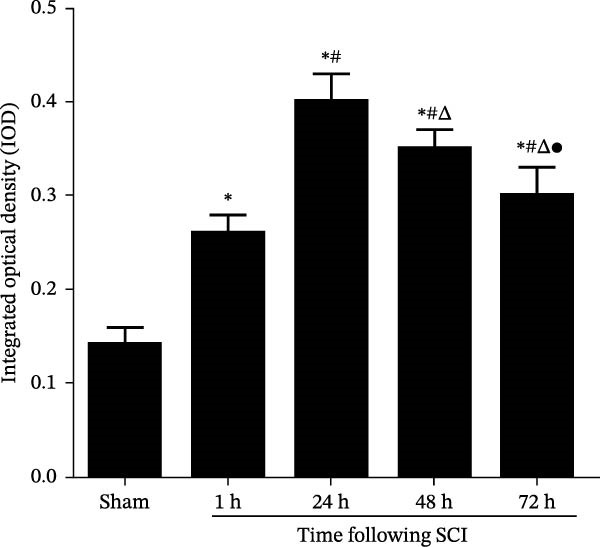
(b)

We apologize for this error.

